# Reimagining HIV service delivery: the role of differentiated care from prevention to suppression

**DOI:** 10.7448/IAS.19.1.21484

**Published:** 2016-12-01

**Authors:** Anna Grimsrud, Helen Bygrave, Meg Doherty, Peter Ehrenkranz, Tom Ellman, Robert Ferris, Nathan Ford, Bactrin Killingo, Lynette Mabote, Tara Mansell, Annette Reinisch, Isaac Zulu, Linda-Gail Bekker

**Affiliations:** 1 International AIDS Society, Cape Town, South Africa; 2 Médecins Sans Frontières, Cape Town, South Africa; 3 World Health Organization, Geneva, Switzerland; 4 Bill & Melinda Gates Foundation, Seattle, WA, USA; 5 United States Agency for International Development, Washington, DC, USA; 6 Centre for Infectious Disease Epidemiology and Research, University of Cape Town, Cape Town, South Africa; 7 International Treatment Preparedness Coalition, Nairobi, Kenya; 8 AIDS Rights Alliance of Southern Africa, Cape Town, South Africa; 9 The Global Fund to Fight AIDS, Tuberculosis and Malaria, Geneva, Switzerland; 10 Centers for Disease Control and Prevention, Atlanta, GA, USA; 11 Desmond Tutu HIV Centre, University of Cape Town, Cape Town, South Africa

The recently updated World Health Organization (WHO) consolidated guidelines on the use of antiretroviral therapy (ART) recommending to “treat all” mark a paradigm shift in the delivery of HIV treatment: from *who* is eligible and *when* to start ART, to *how* to provide client-centred and high-quality care to all people living with HIV (PLHIV). As part of this shift, the new guidance includes service delivery recommendations based on a “differentiated care framework” [1]. Yet, despite the increased global attention paid to differentiated care [2–4], the concept is not well defined.

There is broad agreement that a “one-size-fits-all” model of HIV services will not succeed in providing sustainable access to ART and support services for the 37 million PLHIV today. Instead, health systems will need to both accelerate ART initiation and support retention and viral suppression, which requires adapting HIV services to specific client populations and contexts [5]. Past discussions have looked at differentiated care through a health system's lens – focusing on what aspects of care are needed, how often they are needed, where care should be delivered and who will provide it [6]. An approach to HIV testing, care and treatment that distinguishes client groups according to broad definitions, however, is more likely to succeed.

Differentiated care is a client-centred approach that simplifies and adapts HIV services across the cascade, in ways that both serve the needs of PLHIV better and reduce unnecessary burdens on the health system. Differentiated care incorporates concepts such as simplification, task shifting and decentralization, which have also been called “community-based care, optimized care, patient-centred/focussed care, needs-based care [and] tiered care” [6]. The health system implications of this client-centred approach are clear: when a health system adopts a more responsive model of care, tailored to the needs of various groups of PLHIV, it can allocate resources more effectively, provide better access for underserved populations and deliver care in ways to improve quality of care and life. While differentiated approaches are often more cost-effective in an environment where funding for HIV is under threat, it is critical to ensure that the primary focus for differentiating care remains to improve quality rather than to prop up a misleading “more with less” agenda.

**Figure 1:**
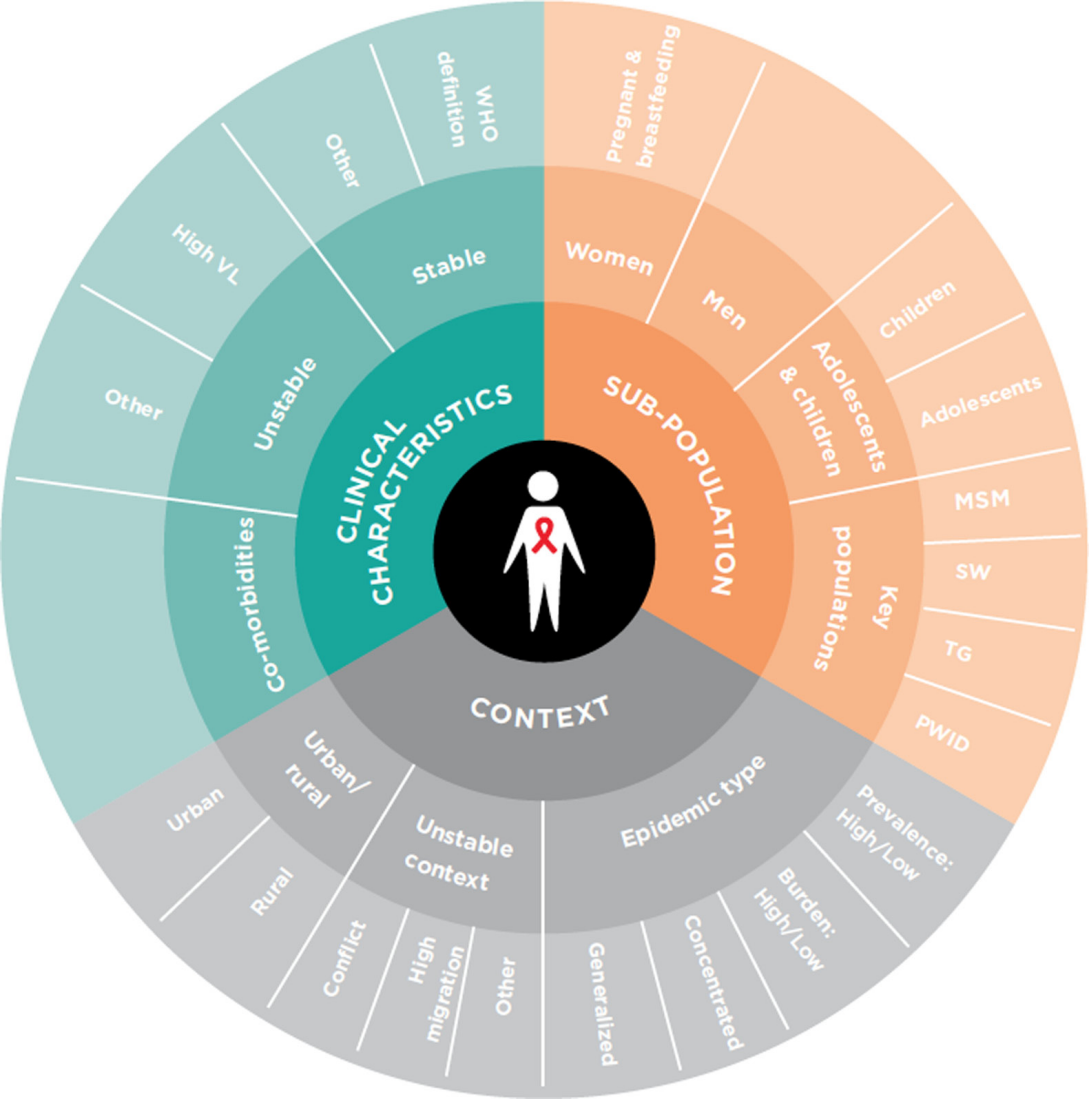
Beyond stable clients: service delivery should be differentiated considering three elements [11].

Well-known models of differentiated care have focused on ART delivery to clients who are clinically stable and have largely been implemented in high-prevalence countries in sub-Saharan Africa. Examples include client-managed groups (e.g. community adherence groups in Mozambique [7]), health care worker-managed groups (e.g. adherence clubs in South Africa [8]), facility-based individual delivery (e.g. “fast track” ART refills in Malawi [9]) and out-of-facility individual delivery (e.g. community drug distribution points in Uganda [10]). To succeed, however, differentiated care must not be limited to stable client models or solely to ART delivery. Policymakers and implementers should “differentiate” care for defined groups according to three elements as defined in Figure 1: (1) clinical characteristics; (2) sub-population; and (3) context [11]. Examples of differentiated care can be found across the cascade and the three elements including expanded PrEP access for sex workers in South Africa [12], a “one-window” approach for people who use drugs in Ukraine [13], targeted peer-led testing of key populations in Thailand [14] and in low-prevalence settings with stable client delivery models in Myanmar [15].

Differentiated care is also a rights-based approach that can act as a modality of stigma and discrimination reduction irrespective of whether or not those rights are formally recognized in laws [16]. By considering the context of the client and health system, differentiated care can help to address policy barriers related to who can dispense versus distribute ART and who can conduct HIV testing. In addition, implementation, particularly at the national level, affords significant opportunities to confront legal and structural barriers that prevent underserved client groups from accessing services [17]. While national policies endorsing differentiated care are necessary for scale-up of HIV services, successful implementation will be dependent on an enabling environment inclusive of a robust drug supply (including fast tracked drug pick-ups and 3–6 month ART refills); access to laboratory monitoring, in particular viral load; a reliable monitoring and evaluation system; and recognition of lay workers. Achieving and sustaining these high-quality services also requires an empowered PLHIV community and civil society. Together, these bodies can advocate and create demand for services that are best tailored to the needs of clients in a given context.

The release of the new WHO guidelines add to the momentum around differentiated care, as evidenced by PEPFAR's Technical Considerations and the Global Fund's toolkit [3, 4] and provide opportunities to reimagine, reorganize and scale up client-centred approaches to HIV service delivery at the national level [1]. The inclusion of differentiated care also catalyses long-standing efforts of rights and community advocates to provide holistic and supportive care, particularly to underserved client groups [18].

Thirty-seven million PLHIV worldwide need lifelong ART. To achieve this, countries must adopt and adapt existing models of differentiated care to meet both the diverse needs of PLHIV and the capacity and constraints of their health systems. To ensure sustainability, successful programmes must be supported by national policies and be adequately funded. The impact of the scale-up of differentiated care models should be evaluated with clear indicators, including quality and outcomes of care, client and health care worker satisfaction, and costs to both the client and the health system. As the models are implemented and improved through analysis of programme data, quality improvement mechanisms and implementation research, stakeholders can work together to address the priority challenges that arise.

Differentiated care is not just about stable clients – but providing quality care from prevention to suppression, including for clients who are unstable or have advanced disease. The global HIV community must seize the opportunity to reimagine service delivery where focus is placed on the quality of services that PLHIV receive. As has been demonstrated throughout the history of the HIV response, lessons learned from HIV can inform and improve care and service delivery across a range of health issues and vice versa. Hence, leveraging the concept of differentiated care beyond HIV to other chronic diseases for all clients will strengthen health systems and contribute to reaching Sustainable Development Goal 3 – “good health and well-being” [19]. To reach that goal, ministries of health, implementing partners, donors, civil society and communities of PLHIV will first need to unite around a differentiated care concept that puts people at the centre of services.
